# The solubilisation of boar sperm membranes by different detergents - a microscopic, MALDI-TOF MS, ^31^P NMR and PAGE study on membrane lysis, extraction efficiency, lipid and protein composition

**DOI:** 10.1186/1476-511X-8-49

**Published:** 2009-11-11

**Authors:** Ulrike Jakop, Beate Fuchs, Rosmarie Süß, Gudrun Wibbelt, Beate Braun, Karin Müller, Jürgen Schiller

**Affiliations:** 1 Leibniz Institute for Zoo and Wildlife Research, Alfred-Kowalke-Str. 17, D-10315 Berlin, Germany; 2 University of Leipzig, Faculty of Medicine, Institute of Medical Physics and Biophysics, Härtelstr. 16-18, D-04107 Leipzig, Germany

## Abstract

**Background:**

Detergents are often used to isolate proteins, lipids as well as "detergent-resistant membrane domains" (DRMs) from cells. Different detergents affect different membrane structures according to their physico-chemical properties. However, the effects of different detergents on membrane lysis of boar spermatozoa and the lipid composition of DRMs prepared from the affected sperm membranes have not been investigated so far.

**Results:**

Spermatozoa were treated with the selected detergents Pluronic F-127, sodium cholate, CHAPS, Tween 20, Triton X-100 and Brij 96V. Different patterns of membrane disintegration were observed by light and electron microscopy. In accordance with microscopic data, different amounts of lipids and proteins were released from the cells by the different detergents. The biochemical methods to assay the phosphorus and cholesterol contents as well as ^31^P NMR to determine the phospholipids were not influenced by the presence of detergents since comparable amounts of lipids were detected in the organic extracts from whole cell suspensions after exposure to each detergent. However, matrix-assisted laser desorption and ionization time-of-flight mass spectrometry applied to identify phospholipids was essentially disturbed by the presence of detergents which exerted particular suppression effects on signal intensities. After separation of the membrane fractions released by detergents on a sucrose gradient only Triton X-100 and sodium cholate produced sharp turbid DRM bands. Only membrane solubilisation by Triton X-100 leads to an enrichment of cholesterol, sphingomyelin, phosphatidylinositol and phosphatidylethanolamine in a visible DRM band accompanied by a selective accumulation of proteins.

**Conclusion:**

The boar sperm membranes are solubilised to a different extent by the used detergents. Particularly, the very unique DRMs isolated after Triton X-100 exposure are interesting candidates for further studies regarding the architecture of sperm.

## Background

The lipids of the cellular membrane have a significant impact on its mechanical and functional properties. The lipid composition, in particular the cholesterol moiety, the headgroup of phospholipids (PLs) and the number of double bonds in the acyl, alkyl or alkenyl residues are the main determinants of membrane flexibility, its fusion behaviour and the interaction with proteins. This is particularly important in the case of spermatozoa that possess a considerable content of highly unsaturated acyl (or alkyl and alkenyl) residues, in particular docosahexaenoyl (22:6) and docosapentaenoyl (22:5) residues [1]. This and the meticulously regulated cholesterol content [2] are essential for the successful interaction with female genital tract components and the completion of the fertilization-related fusion processes. Consequently, the analysis of cellular (not only spermatozoal) lipids is of increasing importance and terms such as "lipidomics" were recently introduced [3].

In contrast to proteins, however, commonly accepted and standardised protocols of lipid analysis are yet scarcely established [4]. The enrichment of lipids by extraction and the simultaneous removal of polar compounds, usually by the most common organic solvent system chloroform/methanol (CHCl_3_/CH_3_OH) according to Bligh and Dyer [5], are normally considered as the first step of lipid analysis. Detergents are also often used for the solubilisation of lipids from biological tissues or cells because they provide an important advantage [6]: Because no (hazardous) organic solvents are required, protein analysis can be simultaneously performed from the same extracts. Moreover, it has been shown that certain parts of biological membranes can be discriminated by their different solubilities in detergents. So-called "detergent-resistant membrane domains" (DRMs) that are rich in cholesterol and sphingomyelin attract considerable attention because they contain GPI-anchored membrane proteins which often are involved in cellular signalling or protein trafficking [7,8]. The lower densities of these lipid-protein complexes with less incorporated detergent allow their isolation from other membrane parts by flotation on density gradients. At present, it is still under discussion whether those DRMs reflect real organisational units of cellular membranes or represent only an artificial clustering upon their preparation with detergents.

The preparation of DRMs from spermatozoa has been described for several mammalian species such as mouse and Guinea pig [9], human [10], pig [11] and cattle [12]. Sperm DRMs were so far exclusively isolated by the use of Triton X-100. Only Ermini *et al*. [13] used Brij 98 in the case of human leukocytes and sperm membranes. However, many different detergents were applied to the extraction of cellular lipids and - with variable outcome - for DRM preparations from other cell types. Schuck *et al*. [14] showed on the example of MDCK and Jurkat cells that the solubilisation by different detergents may reflect different aspects of membrane architecture regarding proteins and lipids as well. Detergents can be differentiated into many subclasses (e.g. strongly ionic detergents, able to form hydrogen bonds (sodium cholate), hydrophobic detergents with long polyoxyether chains (Brij or Tween 20) and aromatic rings (Triton X-100), zwitterionic detergents (CHAPS), etc.) and the use of each of them confers individual advantages and drawbacks [6]. Based primarily on the optimization of their protein-solubilizing properties, most detergents are (despite their largely differing "critical micelle concentrations" (cmc)) typically used in the range of 0.5-2% for practical purposes [6].

As the detergent-based solubilisation of membranes has been developed to a main instrument for the isolation of lipids, proteins and functional membrane domains, we (i) aimed to analyse the solubilisation of boar sperm membranes by different detergents. Unfortunately, detergents exhibit a considerable disadvantage in comparison to volatile organic solvents: Their complete removal is much more difficult. Due to their amphiphilic properties, time-consuming chromatographic techniques are necessary in order to remove detergents, whereas common solvents such as CHCl_3 _can be easily evaporated under reduced pressure. Therefore, we (ii) wanted to ensure that the presence of enclosed detergents has no deleterious influence on the methods typically used for the analysis of the solubilised membrane material including isolated DRMs. An organic extraction after pre-treatment of spermatozoa with various detergents revealed very similar quantitative yields of phospholipids and cholesterol. This result will be confirmed by biochemical methods and ^31^P NMR as well.

With the invention of soft ionization mass spectrometry (MS), lipid mixtures are often analysed by this highly sensitive method [15] the application of which would be preferable for small amounts of DRM material. However, it is well known that MS detection is strongly affected by the presence of impurities, for instance, salts, buffer constituents as well as detergents. This particularly holds for electrospray ionization that is the nowadays most common ionization method used for lipid analysis [16] but is also a problem in the case of matrix-assisted laser desorption and ionization time-of-flight (MALDI-TOF) MS that is increasingly used for the analysis of lipids [17]. Therefore, it is rather surprising that the influence of different detergents on the MALDI-TOF MS detectability of lipids and the best method of detergent removal was only studied for cardiolipin that is, however, a less common PL [18]. In contrast, there are many studies on the mass spectrometric detectability of proteins in the presence of detergents [19] and optimum methods of detergent removal from the aqueous phase [20] have been established.

This study demonstrates that residual amounts of detergents - which even remain in DRM lipids subsequent to extraction with organic solvents - essentially disturb the analysis of membrane lipids by MALDI-TOF MS. Therefore, analyses of solubilised boar sperm membranes investigated in this study were preferentially performed by biochemical methods and ^31^P NMR. First, we observed by light and electron microscopy which membrane areas of boar spermatozoa were actually affected by different detergents at 4 and 38°C. Subsequent to mechanical detachment of disintegrated sperm membranes and removal of cellular debris by centrifugation, we quantified the amount of sperm lipids in the supernatant. Furthermore, we isolated DRMs by ultracentrifugation of supernatants over sucrose density gradients. In dependence of the detergent, this study revealed very different solubilisation patterns and different efficiencies to obtain visible DRM bands from boar spermatozoa. We could also show that only membrane solubilisation by Triton X-100 leads to an enrichment of cholesterol, sphingomyelin, phosphatidylinositol and phosphatidylethanolamine in visible DRM bands accompanied by the selective accumulation of proteins.

## Materials and methods

### Chemicals

All detergents (cf. Additional file 1, Table S1) were commercially available and used as supplied. They were dissolved in HEPES-buffered salt solution (HS): 150 mM NaCl, 5 mM HEPES (pH 7.4) to give double concentrated detergent solutions compared to the final concentration given in Additional file 1, Table S1 (first column). Pluronic F-127 was dissolved in dimethylsulphoxide (200 mg/ml) and subsequently diluted 1:5 with HS.

The chemical structures of all detergents used in this study are listed in Additional file 1, Table S1 together with their formulas, their molecular masses and their chemical structures. As the detergents with ethylene-glycol repeat units are characterised by a considerable distribution of their masses, only the mass of the dominating compound (cf. Results Section) is provided. Please note that this compound is not always in agreement with the information provided by the detergent manufacturer. For instance, it turned out from the mass spectra that the Tween 20 represents actually a mixture of different compounds. This is in agreement with a previous study by Ayorinde [21]. As this is, however, science of its own, this aspect is not emphasized to a larger extent in this study.

All phospholipid standards used as reference compounds and for model experiments were obtained from Avanti Polar Lipids (Alabaster, AL, USA) as 10 mg/ml solutions in chloroform (CHCl_3_) and used without further purification. Cholesterol was from Fluka. All further chemicals for sample preparation, MALDI-TOF MS (2,5-dihydroxybenzoic acid (DHB)) as well as all solvents (chloroform and methanol) were obtained in highest commercially available purity from Fluka (subcontractor of Sigma-Aldrich Chemie GmbH, Taufkirchen, Germany) and were also used as supplied. Glutaraldehyde, EPON 812, lead citrate, osmium tetroxide and uranyl acetate were purchased from Serva (Heidelberg, Germany).

### Spermatozoa - treatment with detergents and preparation of DRMs

Boar semen was obtained from different fertile animals of a breeding station (Hermitage Deutschland GmbH Besamungseberstation, Golzow, Germany). Aliquots (about 40 ml) of fresh ejaculates from four males were centrifuged (8 min, 700 × g, 22°C). After removal of seminal fluid, the sperm pellet was re-suspended in 40 ml HS and centrifuged again. The supernatant was discarded and the sediments used for further experiments. Sperm concentration was adjusted with HS to about 1.7 × 10^9 ^cells per ml and one aliquot was frozen at -80°C.

Other aliquots of this cell suspension were mixed with an equal volume of the double concentrated, pre-cooled (4°C) detergent solutions or an equal volume of HS without detergent (control) and incubated for 30 min at 4°C. Afterwards the samples were homogenised in a glass Dounce homogeniser by 10 strokes on ice. The obtained suspension was centrifuged (5 min, 1300 × g, 4°C) to pellet cellular debris. The volumes of pellet and corresponding supernatant were noted and aliquots were frozen at -80°C for further analyses. One aliquot of each supernatant was mixed with an equal volume of 80% sucrose in HS and carefully transferred under a continuous sucrose gradient (20-40% in HS). Ultracentrifugation (18 h, 100 000 × g, 4°C) was performed in a SW 40 Ti rotor of a XL-70 ultracentrifuge (Beckman Instruments GmbH, Munich, Germany). All visible bands were documented and collected by use of a syringe with a bended needle. 1 ml of the bottom fraction was also collected and all these samples were divided and frozen at -80°C until subsequent extraction and analysis. In some cases, the whole gradient (about 14 ml) was collected in 1 ml steps to analyse the optical density and the cholesterol content.

### Lipid extraction by organic solvents

Lipid extraction was performed according to Bligh & Dyer [5] with slight modifications: 1.5 ml chloroform/methanol (CHCl_3_/CH_3_OH, 1:2, v/v) was added to 0.4 ml sperm, membrane or DRM suspension. Samples were thoroughly mixed and incubated for 30 min at 22°C. 0.5 ml CHCl_3 _was added and samples were vortexed for 0.5 min. 0.5 ml 40 mM acetic acid was added and samples vortexed again. In agreement with a previous study, no hydrolysis was induced by the addition of moderately concentrated acetic acid although sperm plasmalogens are very sensitive to acids [22]. After phase separation for 20 min at 1000 × g and 4°C, the chloroform phase was carefully collected and the aqueous phase mixed again with 1 ml CHCl_3_. After a second centrifugation step, the chloroform phases were combined and the whole volume was divided into aliquots for the biochemical determination of cholesterol and phosphate as well as for ^31^P NMR and MALDI-TOF analyses.

### Biochemical determinations of cholesterol and phosphate

Cholesterol concentrations were determined by using a commercially available cholesterol test kit (E013905, r-biopharm, Darmstadt, Germany) based on the generation of H_2_O_2 _upon cholesterol oxidation under catalysis of the enzyme cholesterol oxidase. Samples and cholesterol standards were dissolved in isopropanol, treated as described in the manual of the test kit and the colour development was quantified on a photometer UV A160 (Shimadzu) at 405 nm. Cholesterol concentrations in DRM bands were - without prior extraction with organic solvents - additionally determined by a fluorescence-based enzyme assay (Amplex Red Cholesterol Assay Kit A12216, Invitrogen GmbH, Karlsruhe, Germany).

The phosphate content was determined according to [23]. 400 μl perchloric acid were added to each sample and a separate phosphate standard. All samples were boiled up to 120 min at 180°C until the solutions became colourless. 4 ml molybdate solution (2.2 g (NH_4_)_6_Mo_7_O_24 _× 4 H_2_O and 14.3 ml H_2_SO_4 _in 1 l distilled water) and 500 μl 10% (w/v) ascorbic acid were added to the cooled samples with subsequent vortexing. After boiling for 10 min and cooling down again in ice water, the colour development was monitored on a photometer UV A160 (Shimadzu) at 812 nm.

### MALDI-TOF mass spectrometry

Total organic extracts of detergent-treated or control boar spermatozoa as well as the isolated detergents and the model lipid systems ± detergents were investigated by MALDI-TOF MS. A 0.5 M 2,5-dihydroxybenzoic acid (DHB) solution in methanol [24] was used in all cases. A recent investigation has shown that this isomer of dihydroxybenzoic acid is most suitable as MALDI matrix [25]. Although trifluoroacetic acid (TFA) is often added as a cationising agent, this was not done here as plasmalogens that are essential constituents of boar spermatozoa lipids are extremely sensitive towards even traces of strong acids (such as TFA) and decompose rapidly under acidic conditions to give the corresponding lysolipids [22].

All MALDI-TOF mass spectra were acquired on a Bruker Autoflex mass spectrometer (Bruker Daltonics, Bremen, Germany). The system utilizes a pulsed nitrogen laser, emitting at 337 nm. The extraction voltage was 20 kV and gated matrix suppression was applied to prevent the saturation of the detector by matrix ions [26]. 128 single laser shots were averaged for each mass spectrum. The laser fluence was kept about ten percent above threshold (i.e. the minimum laser fluence required to detect any signals) to obtain an optimum signal-to-noise (*S/N*) ratio. In order to enhance the spectral resolutions all spectra were acquired in the reflector mode using delayed extraction conditions. A more detailed methodological description of MALDI-TOF MS is provided in [4] and [27].

In selected cases PSD (post source decay) experiments were also performed according to [28] in order to clarify the fatty acyl compositions of the lipid species and the positions of the individual fatty acyl residues.

### High resolution ^31^P NMR spectroscopy

The dried organic spermatozoa extracts were solubilised according to [29,30] in 50 mM TRIS (pH 7.65) containing 200 mM sodium cholate and 5 mM EDTA. After vortexing, ^31^P NMR spectra were recorded on 0.5 ml samples in 5 mm NMR tubes on a Bruker DRX-600 spectrometer operating at 242.88 MHz for ^31^P. All measurements were performed using a selective ^31^P NMR probe at 37°C with composite pulse decoupling (Waltz-16) to eliminate ^31^P - ^1^H coupling. Pulse intervals of the order of T_1 _were used to allow quantitative analysis of PL integral intensities [31].

Other NMR parameters were as follows: acquisition time: 1 s, data size: 8-16 k, 60° pulse (7 μs), pulse delay 2 s and a line-broadening (LB) of 1 Hz. Chemical shifts were referenced by using the resonance of phosphatidic acid (PA) 14:0/14:0 (DMPA) that was added to all samples in an amount of 10 μg (corresponding to 32.5 μmol/l) as concentration and frequency standard.

Spectra were processed using the software "1D WIN-NMR" version 6.2^® ^(Bruker Analytische Messtechnik GmbH, Rheinstetten) including the deconvolution (II) routine for peak area determination.

### Microscopic evaluation of membrane solubilisation by detergents

For the evaluation of sperm morphology by light microscopy, 100 μl of detergent-treated samples or the corresponding control were fixed with 500 μl of 0.5% formol solution in HS. The incubation of sperm cells with the different detergents was performed at 4°C as well as at 38°C. The solubilisation of sperm head membranes was observed on wet slides under phase contrast optics at a 100-fold magnification (Axioskop, Zeiss, Jena, Germany). 200 cells per sample were divided into three categories: intact, detaching but still adherent and completely detached head membranes over the acrosome. Pictures were recorded with a camera MC80 (Zeiss, Jena, Germany) at a magnification of 500.

For transmission electron microscopy, 2 ml detergent-treated or control sperm suspensions were centrifuged (8 min, 1000 × g, 4 or 22°C, respectively). The supernatant was removed and the pellet was fixed in 100 μl 3% glutaraldehyde solution (w/v). After washing in Dulbecco's phosphate buffered salt solution (pH 7.2) samples were fixed again in 2% osmium tetroxide. Dehydration was performed in ethanol and samples were embedded in EPON 812 before preparation of ultrathin sections and staining with uranyl acetate and lead citrate. Ultrathin sections were examined with a TEM Zeiss 902A electron microscope (80 kV; Oberkochen, Germany).

### Protein determination and PAGE

The protein content of all samples was determined by BCA protein assay as described in [32]. Samples (5 μg per lane) were separated on 20% SDS-PAGE (Mini Protean III; Bio-Rad, München, Germany) and the gel was silver-stained according to [33].

## Results

### Boar sperm lipid extraction and analysis in the presence of detergents

The focus of this paper is the qualitative and quantitative investigation of sperm lipids (phospholipids and cholesterol) subsequent to treatment of the cells with different detergents (see Additional file 1, Table S1). To that purpose, the extraction efficiency of the established Bligh and Dyer method [5] towards detergent-treated sperm samples (without further membrane separation steps) was tested and compared to a control without any detergent (w/o). As measures of the extraction efficiency, the cholesterol and phosphate (corresponding to the phospholipid) contents in the organic sperm extracts were determined by biochemical assays. To avoid the potential contribution of phosphate from the seminal fluid, a completely phosphate-free buffer was used to remove seminal fluid by repeated washing steps of fresh semen.

**Figure 1:**
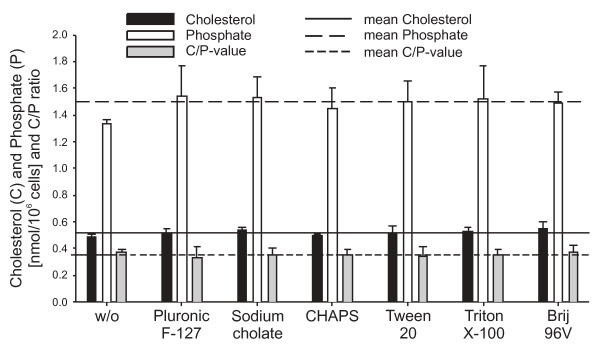
**Amounts of phosphate (as the measure of phospholipids) and cholesterol determined by biochemical assays in the organic extracts of detergent-treated boar spermatozoa**. From these data, the ratio between cholesterol and phosphate (C/P) was also calculated. Sperm cells were incubated with detergents for 30 min at 4°C and lipids were extracted and determined as described in the Materials and Methods section. The mean ± SD of two different extractions per sample is shown. Please note that there are only slight differences between the individual detergents.

The variation coefficients for repeated measurements (n = 4) of a standard cholesterol or phosphate solution are 6% and 3%, respectively. The variation coefficients of cholesterol and phosphate determinations after repeated extractions of the same sperm suspension (n = 6 aliquots) are 7% and 3%, respectively (data not shown). For the detergent-treated sperm cells, two extractions per sample were performed and the mean values are shown in Fig. 1. In the presence of detergents, averages of 0.52 ± 0.02 nmol cholesterol and 1.52 ± 0.06 nmol phosphate were determined per 10^6 ^spermatozoa. This is comparable to the control sample resulting in 0.49 nmol cholesterol and 1.33 nmol phosphate per 10^6 ^spermatozoa. Nevertheless, an enhanced phosphate and cholesterol content could be determined in the detergent-treated samples. This indicates that the extractability of lipids is slightly facilitated by the presence of detergents. The mean C/P (cholesterol/phosphate ratio) in the organic extracts of the detergent-treated spermatozoa was 0.35 ± 0.02 compared to 0.37 in the control. The variation coefficients for cholesterol (4.2%) and phosphate (3.8%) contents in detergent-treated samples were in the same range as the accuracy of repeated extractions.

**Figure 2:**
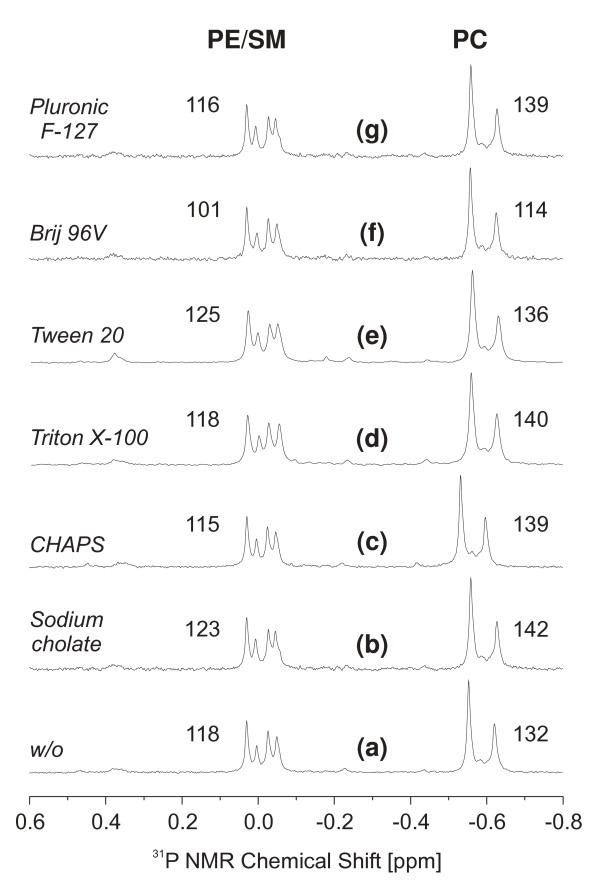
**^31^P NMR spectra of the organic extracts of detergent-treated boar spermatozoa**. Sperm cells were incubated with the indicated detergents for 30 min at 4°C and phospholipids were extracted as described in the "Materials and Methods" section. Individual samples (organic extracts) were evaporated to dryness and subsequently re-dissolved in aqueous sodium cholate according to (31). Sodium cholate is the detergent of choice because it prevents formation of supramolecular phospholipid aggregates and forms very small micelles that are characterized by high mobility and provide, therefore, highly resolved ^31^P NMR spectra. The numerical values correspond to the amount of PL [nmol] detected in the individual samples and were calculated in comparison to a known amount of PA 14:0/14:0 (not shown) that was added in a final concentration of 10 μg per sample. Abbreviations used in peak assignments: SM, sphingomyelin; PC, phosphatidylcholine; PE, phosphatidylethanolamine. The applied detergents are explicitly given in the different traces.

In order to exclude that the different detergents interfere with the applied biochemical assays, the phospholipid contents of the individual spermatozoa samples were additionally assessed by ^31^P NMR spectroscopy and the corresponding spectra are shown in Fig. 2. All resonances were scaled with reference to phosphatidic acid (PA 14:0/14:0) as concentration standard that is not shown in Fig. 2 as it appears at much lower field. The numerical values given in Fig. 2 correspond to the nanomoles of phosphatidylethanolamine (PE) and sphingomyelin (SM) on the one hand and phosphatidylcholine (PC) on the other hand in the individual samples and it is evident that there are only slight differences in dependence of the applied detergents. A more detailed data evaluation will be performed below. Please note that the qualities of the individual spectra are comparable. This clearly indicates that any residual detergent does not have a strong impact on the achievable resolution. It could also be shown (data not shown) that ^31^P NMR spectra can be even recorded from the native detergent extracts and there is no absolute need to re-extract these solutions with organic solvents. However, all samples were planned to be also characterized by MALDI MS that clearly requires solvent extraction. In order to use the same sample for ^31^P NMR as well as MALDI MS, all samples were re-extracted with organic solvents.

Despite smaller deviations, the ^31^P NMR data confirm the quantitative spectrophotometric results: There are no significant differences between the individual spectra and this clearly indicates that all applied detergents extract the sperm phospholipids to a very similar extent. It is also evident that the ratio between the individual phospholipid classes [34] is not altered in the presence of different detergents.

**Figure 3:**
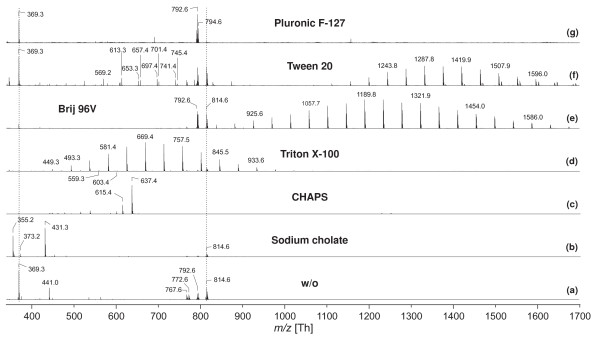
**Positive ion MALDI-TOF mass spectra of the organic extracts of detergent-treated boar spermatozoa**. Sperm cells were incubated with detergents for 30 min at 4°C and lipids were extracted as described in Materials and Methods. The organic extracts were directly used for MALDI-TOF MS. Selected peaks are labelled according to their *m*/*z *ratios. The dotted vertical lines indicate the characteristic spermatozoa lipids at *m*/*z *= 369.3 (cholesterol) as well as 792.6 and 814.6 (GPC_Plasm_). The applied detergents are explicitly given in the different traces. For further details see Materials and Methods section.

In order to analyse the qualitative lipid composition of detergent-treated boar spermatozoa in more detail, MALDI-TOF MS was additionally used because it is a fast and convenient method to characterise organic cell extracts [34]. The extraction of sperm lipids by organic solvents subsequent to the detergent treatment leads to the removal of salts, buffers and other polar components such as proteins. Unfortunately, however, significant amounts of detergent remain in the organic phase [18]. This is obvious from the positive ion MALDI-TOF mass spectra corresponding to the different detergent treatments that are shown in Fig. 3.

The mass spectrum of the pure organic extract from boar spermatozoa (control in HS) is shown as reference (**3a**). The lipid composition of boar spermatozoa is quite simple [34] and much less components are detectable than in the case of, for instance, human spermatozoa [35]. This is one additional reason why this study is focused exclusively on boar spermatozoa. Although boar spermatozoa contain also significant amounts of PE and seminolipid [34], the PC and the cholesterol contents will be exclusively considered in this study from two reasons: (a) both lipids are very abundant in spermatozoa and (b) less sensitively detectable lipids such as PE may be suppressed by signals of lipids with quaternary ammonia groups (e.g. PC and LPC) [26]. However, cholesterol and PC are very sensitively detectable - at least if DHB is used as the matrix.

The peaks at *m/z *= 790.6 and 812.6 (**3a**) can be assigned to the H^+ ^and Na^+ ^adducts of 1-O-palmitenyl-2-docosahexaenoyl-*sn*-glycero-3-phosphocholine (PC plasmalogen), whereas the peaks at *m/z *= 792.6 and 814.6 are stemming from 1-palmityl-2-docosahexaenoyl-*sn*-glycero-3-phosphocholine (PC alkyl ether). Peaks at *m/z *= 794.6 and 816.6 belong to 1-O-palmityl-2-docosapentaenoyl-*sn*-glycero-3-phosphocholine. Additionally, cholesterol is detected at *m/z *= 369.3. This peak corresponds to the H^+ ^adduct of cholesterol subsequent to water elimination [35].

It is evident from the comparison of this control spectrum with the spectra recorded in the presence of detergents that the lipid signals of spermatozoa are significantly influenced by the presence of the residual detergents. All spectra were scaled in the way that the base peak, i.e. the most intense peak possesses the same intensity. In the presence of sodium cholate (**3b**), the intensities of the PC as well as the cholesterol peaks are significantly diminished and the detergent peak (*m/z *= 431.3) is most intense. In the presence of CHAPS (**3c**) there are not even traces of lipid signals anymore. This is particularly remarkable as the excellent signal-to-noise ratio should allow the detection of even very small amounts of lipids. In the presence of Triton X-100 (**3d**) small amounts of spermatozoa lipids (e.g. *m/z *= 814.6) are detectable. However, it is obvious that under these conditions the cholesterol signal (*m/z *= 369.3) is barely detectable. This is remarkable as the comparison with the pure organic extract (**3a**) of boar spermatozoa clearly indicates that the cholesterol peak should be of higher intensity than the PC signals. Therefore, it is likely that different lipid classes are suppressed by the individual detergents to different extents.

Both, Tween 20 (**3f**) and Brij 96V (**3e**) do not significantly affect the detectability of the spermatozoa lipids, although it is obvious that the cholesterol is detected with a much higher intensity in the presence of Tween 20 than in the presence of Brij 96V. Therefore, ionic detergents such as sodium cholate or particularly CHAPS lead to a more marked suppression of lipids than non-ionic detergents. Finally, Pluronic F127 (**3g**) has only a very weak suppression effect on the intensities of the lipids. Although it was not the aim of this work to investigate this effect in more detail, it seems likely that this effect is caused by the comparably high molecular mass of this detergent. It is also unknown why only the H^+ ^but not the Na^+ ^adduct of the PC species is detectable in the presence of Pluronic F127.

It is obvious that all applied detergents can be easily differentiated by MALDI-TOF MS as they provide significantly different spectra. A detailed discussion of the mass spectra of detergents was beyond the scope of this paper. For details see [21,36].

### Detergent-dependent solubilisation patterns of boar sperm membranes

**Figure 4:**
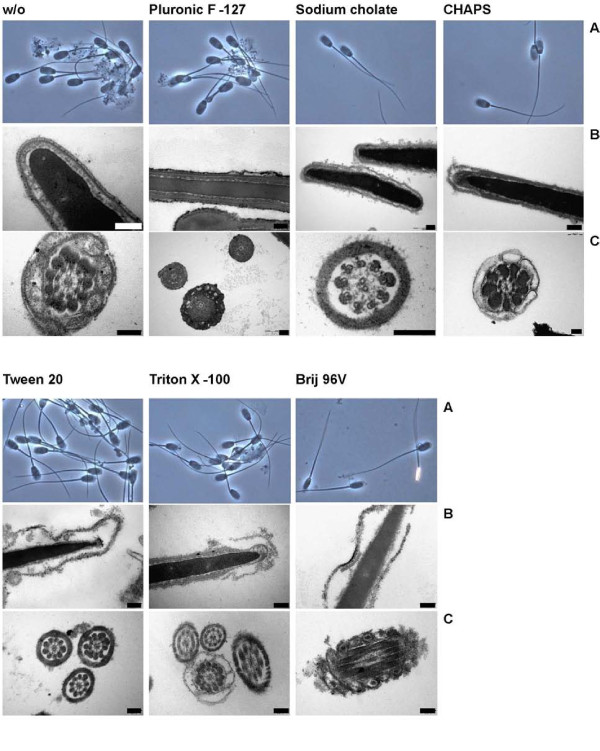
**Morphological patterns of boar sperm membrane lysis after incubation with the indicated detergents for 30 min at 4°C**. Sperm samples were observed by light microscopy (A, phase contrast, magnification of 500) as well as by transmission electron microscopy (B, C). For further details see the Materials and Methods section. Each bar represents 200 nm. The quantitative evaluation of the microscopic investigations is given in Tab. 2.

Provided with the analytical methods to characterise detergent extracts of sperm membranes, we were finally interested in the solubilisation patterns on boar sperm membranes caused by the different detergents (cf. Additional file 1, Table S1). Spermatozoa consist of a head and tail region which both are surrounded by the cellular lipid membrane. The head of mammalian spermatozoa contains in its anterior part the "acrosome", a lysosome-derived organelle with lytic enzymes which are released during the exocytotic process of the acrosome reaction and enable the cell to penetrate the Zona pellucida of the egg. Under light microscopic observation it is possible to evaluate the detachment or removal of the membranes over the acrosomal region. As shown in Table 1 and illustrated in Fig. 4, about 93% of the control sperm cells are intact after 30 min incubation at 38°C. The reduction of the temperature to 4°C for 30 min did not cause a disturbance of the membranes in the absence of any detergent although boar spermatozoa are known to be sensitive to the cold. In the presence of Pluronic F-127 the head membranes appear undisturbed at both temperatures, too. All other detergents, however, lead to a detachment of the head membranes. This effect becomes more pronounced at the higher temperature and at lower hydrophilic-lipophilic-balance (HLB) values of the non-ionic detergents and leads to the most significant detachment of the membranes from the sperm head. Light microscopy also visualizes distinct patterns of membrane detachment. As shown for CHAPS and Triton X-100, a sharp line-shaped detaching membrane is observed. In the case of Tween 20, most of the membranes which detach from the anterior part of the sperm heads appear diffuse and patchy. Note that the membranes are never completely released from the cells by detergents. Therefore, mechanical stripping of detaching membranes is needed to deliver them into the supernatant.

**Table 1:** Percentage (mean ± SD) of boar sperm cells with intact, detaching but still adherent, or completely detached head membranes after incubation with detergents for 30 min at 4°C or 38°C

	intact membranes		detaching membranes		detached membranes	
Detergent	4°C	38°C	4°C	38°C	4°C	38°C
w/o	91,2 ± 8,5	92,7 ± 5,1	8,3 ± 8,6	6,1 ± 3,7	0,4 ± 0,7	1,2 ± 2,3
Pluronic F-127	94,1 ± 4,5	95,3 ± 4,0	5,6 ± 4,7	4,1 ± 3,8	0,3 ± 0,5	1,0 ± 1,2
Sodium cholate	0,3 ± 0,8	0,1 ± 0,4	96,0 ± 3,4	97,1 ± 2,3	3,7 ± 3,1	2,7 ± 2,2
CHAPS	0,3 ± 0,8	0,0 ± 0,0	96,4 ± 5,7	94,7 ± 3,5	3,3 ± 5,8	5,3 ± 3,5
Tween 20	43,1 ± 25,1**	0,0 ± 0,0**	56,0 ± 24,7**	98,0 ± 1,7**	0,9 ± 0,9	2,0 ± 1,7
Triton X-100	0,1 ± 0,3	0,6 ± 0,9	97,2 ± 2,5	96,7 ± 2,0	2,7 ± 2,6	2,8 ± 1,9
Brij 96V	0,1 ± 0,3	0,3 ± 0,5	98,8 ± 1,9*	91,8 ± 7,6*	1,1 ± 1,6*	7,9 ± 7,7*

* significant difference in dependence of temperature (student t-test for paired samples, p ≤ 0,05, n = 9)

** significant difference in dependence of temperature (student t-test for paired samples, p ≤ 0,01, n = 9)

Sperm cells of ejaculates from 9 different boars were treated and fixed as described in the "Materials and Methods" section. 200 cells per sample were counted under phase contrast optics (magnification of 1000).

**Figure 5:**
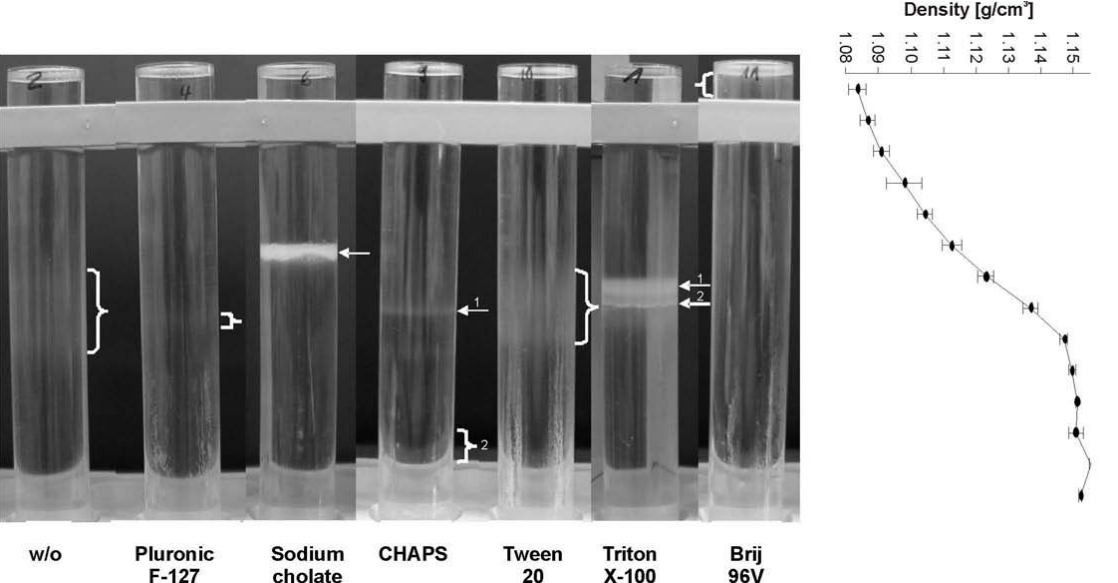
**Bands of detergent-resistant membrane fractions in sucrose gradients after ultracentrifugation**. Boar sperm cells were incubated with detergents for 30 min at 4°C, membrane fractions were mechanically detached and separated from the remaining cell pellet by centrifugation. The supernatant was transferred under a continuous sucrose gradient for subsequent flotation. The obtained density gradient is additionally shown at the right of the figure. For further details see the Materials and Methods section.

Indeed, the higher resolution of transmission electron microscopy (traces "B" and "C" in Fig. 4) gives more details than the light microscopic observations. In the control samples many cells with smooth membrane structures are visible although we also found several cells that (at least partially) lost their membranes. This might have been caused by the washing and dehydration protocol even in the presence of glutaraldehyde. As evidenced by light microscopy, Pluronic F-127 seemed to be able of protecting the membranes of sperm cells. Although a detailed (quantitative) image analysis was beyond the scope of this paper, the membrane surfaces appear markedly roughened in the presence of Pluronic F-127 and covered with fine electron dense remnants of lead citrate. In all other detergent preparations, large amounts of detaching membranes in the heads as well as the tail regions were observed. Again, the extent of solubilisation is reciprocally dependent on the HLB values of the applied non-ionic detergents. The most severe changes were observed for Brij 96 V where solubilisation obviously occurs even within the nuclear membrane. Solubilised membranes appear rough with disintegrating structures visible as fine empty holes admixed with electron dense spots (lead citrate). Only with Tween 20, larger vesicular structures of about 50-200 nm diameter were generated in close proximity to the outer surface. However, independent of the applied detergent, we also found some cells with relatively intact membrane structures (cf. the picture of the Triton X-100-treated cells). This particularly concerns the postacrosomal region whereas membranes of the anterior part of the head were destroyed (not explicitly shown).

From these microscopic data we conclude that the solubilisation efficiency of detergents and the resulting detachment of membranes is very specific and, in non-ionic detergents, reciprocally correlated with the HLB value. Moreover, the sensitivity towards detergents differs between individual sperm cells as well as the affected regions.

In a next step we quantified the amount of lipid and protein released from the boar spermatozoa after detergent treatment and subsequent mechanical stripping of solubilised membranes (Table 2). In accordance with morphological observations, only very small amounts of phospholipids and no cholesterol were detectable in the organic extracts of supernatants of samples without detergent treatment and after treatment with Pluronic F-127. As the amount of phospholipid was determined via phosphate assay, a contamination with some inorganic phosphate can not be completely ruled out. Thus, ^31^P NMR studies were also performed to overcome these disadvantages (see below). Tween 20 releases only little lipid material from the cells. This is consistent with the microscopic observation of 43% intact cells after incubation with Tween 20 at 4°C. However, the protein content in the supernatant was comparably high and similar to treatments with other detergents. Triton X-100 and Brij 96V as non-ionic detergents with low HLB values released more membranous material into the supernatant. Repetitions with three ejaculates from different boars revealed 55.8 ± 14.8% released cholesterol and 66.9 ± 3.6% released phospholipid. The most marked destruction of membranes was observed if the zwitterionic CHAPS and anionic sodium cholate were used. Both, cholesterol and phospholipids were released from the cells to a rather similar extent in the presence of these detergents.

**Table 2:** Relative amounts of cholesterol and phospholipid that were released from boar spermatozoa into the supernatant after detergent solubilisation, mechanical treatment and centrifugation

	Release into supernatant after detergent and mechanical treatment			Bottom fraction related to the underlayed amount from supernatant			Band fractions 1/2 related to the underlayed amount from supernatant		
Detergent	Cholesterol	Phospholipid	Protein	Cholesterol	Phospholipid	Protein	Cholesterol	Phospho-lipid	Protein
	[%]	[%]	[mg/10^9 ^cells]	[%]	[%]	[%]	[%]	[%]	[%]
**w/o**	0.0	5.2	0.49		1.7	34.6		49.0	13.7
**Pluronic F-127**	0.0	2.3	0.68		1.9	13.7		10.2	0.0
**Sodium cholate**	79.8	79.0	3.07	4.5	5.4	20.0	36.4	68.6	29.3
**CHAPS**	88.5	85.7	2.57	10.0	20.0	24.6	0.8/11.3	1.0/19.0	0.0/21.0
**Tween 20**	0.0	9.1	2.01		4.2	15.9		38.3	5.8
**Triton X-100**	50.4	68.9	2.28	8.0	8.4	18.3	11.4/10.5	9.8/7.9	0.0/4.7
**Brij 96V**	61.5	61.7	2.27	3.3	2.6	20.8	1.7	2.5	3.8

The sum of the amounts of cholesterol and phospholipid in the pellet and the supernatant is considered as 100%. Additionally, total amounts of protein released into the supernatant (per 10^9 ^cells) and relative amounts of cholesterol, phospholipid and protein in the visible bands and bottom fractions from sucrose gradients after ultracentrifugation are also given. For further details see Materials and Methods section.

### Detergent-dependent isolation of DRMs from boar sperm membranes

In order to isolate DRMs from boar sperm membranes the supernatants of solubilised membranes obtained after detergent treatment and mechanical stripping were transferred under a continuous sucrose gradient. Fig. 5 shows that only sodium cholate and Triton X-100 produced sharp and clearly visible bands after ultracentrifugation at approximate densities of 1.112-1.117 and 1.127-1.138 g/cm^3^, respectively. In the case of Triton X-100, one broader (1) and one smaller (2) band could be discriminated. CHAPS produced two faint bands, the most prominent of which (2, not clearly observable due to the presence of the tube holder) floated just above the bottom fraction (about 1.15 g/cm^3^). Brij 96V gives one faint band at lowest density (< 1.085 g/cm^3 ^above the bar of the tube holder). Tween 20 shows a very broad and faint area from 1.123-1.147 g/cm^3^. A similar region can hardly be identified in the sample without detergent treatment and there is also only a very weak band in the Pluronic F-127-treated sample.

As shown in Table 2, only the sperm supernatants after treatment with sodium cholate, CHAPS, Triton X-100 and Brij 96V contained significant amounts of cholesterol and phospholipids. Among those, only sodium cholate, CHAPS and Triton X-100 accumulated essential amounts of lipids in the band fractions, which are also summarized in Table 2. Particularly after Triton X-100 treatment, slightly more cholesterol than phospholipid was accumulated in both prominent bands. Repetitions with three ejaculates from different boars revealed 28.3 ± 9.0% and 19.4 ± 2.3% of the total cholesterol and phospholipid, respectively, in the band fractions (sum of both). The bottom fraction (1 ml) contained 11.1 ± 4.3% and 9.2 ± 1.2% of the total cholesterol and phospholipids, respectively. In CHAPS-treated samples, most lipids and proteins accumulated in the band above the bottom fraction. In sodium cholate- and CHAPS-treated samples, more protein accumulated in the bands than in the Triton X-100-treated samples, where significant protein content was exclusively found in band 2.

**Figure 6:**
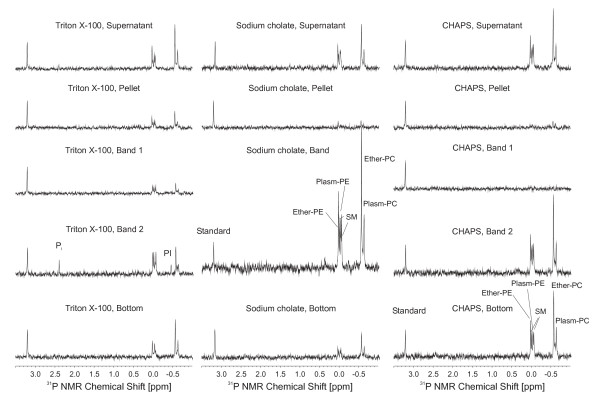
**^31^P NMR spectra of the organic extracts from detergent-solubilised sperm phospholipids subsequent to flotation on a sucrose gradient by ultracentrifugation**. Assignments are given in the individual traces and were made by comparison with the chemical shifts of samples of known compositions. Please note that all spectra are scaled in the way that the resonance of the standard (PA (14:0/14:0) at 3.2 ppm) possesses the same intensity. Please also note the enrichment of PI and SM in the Triton X-100 samples.

As the remaining traces of detergents in the DRMs affect the lipid analysis by MALDI-TOF (spectra not shown, cf. Fig. 3) we finally applied ^31^P NMR spectroscopy for the quantitative phospholipid analysis. Only the organic extracts of the most prominent band fractions after treatment with sodium cholate, CHAPS and Triton X-100 contained enough material to give spectra of reasonable quality as seen in the overview provided in Fig. 6 and the quantitative data given in Table 3.

**Table 3:** Molar concentrations of different phospholipid species in the indicated fractions

	Ether-PE	SM	Plasm-PE	PI	Ether-PC	PC-Plasm	PE:SM:PC
Triton X-100, Supernatant	24.8	29.1	14.4	---	63.0	26.9	**44:32:100**

Triton X-100, Pellet	10.6	18.2	3.4	---	22.3	13.7	**39:51:100**

Triton X-100, Band 1	22.2	45.2	17.2	14.8	43.0	14.9	**68:78:100**

Triton X-100, Band 2	8.2	15.6	4.4	traces	16.3	6.1	**56:70:100**

Triton X-100, Bottom	14.9	14.8	13.6	---	36.0	23.1	**48:25:100**

Sodium cholate, Supernatant	21.2	51.6	9.1	---	59.8	35.8	**32:54:100**

Sodium cholate, Pellet	8.2	traces	traces	---	12.5	4.2	**49:0:100**

Sodium cholate, Band	79.0	95.7	56.9	traces	209.4	57.5	**51:36:100**

Sodium cholate, Bottom	8.5	13.6	3.7	---	24.1	12.6	**33:16:100**

CHAPS, Supernatant	30.8	39.2	17.0	---	66.3	32.7	**48:40:100**

CHAPS, Pellet	traces	traces	traces	---	12.1	6.5	**n.d**.

CHAPS, Band 1	traces	traces	traces	---	traces	traces	**n.d**.

CHAPS, Band 2	traces	traces	traces	---	traces	traces	**n.d**.

CHAPS, Bottom	34.5	58.9	22.9	traces	88.9	43.0	**44:45:100**

All concentrations were determined by ^31^P NMR relative to PA (14:0/14:0) that was present in all cases in a final concentration of 32.5 μmol/l (cf. Fig. 6). Please note that all data cannot be given with an estimated accuracy higher than ≤ 15%. This is due to the relatively small concentrations, the low sensitivity of NMR and the partial overlap between the individual species. This particularly concerns the PE species and SM. The last column gives the calculated intensity ratio between PE, SM and PC without further differentiation. PC was arbitrarily set as 100% in all cases. For details see text.

It is already evident from the comparison of the individual spectra that the PL content differs significantly between the different fractions. Please note that the resonance at 3.20 ppm corresponds to PA (14:0/14:0) that was added to each sample in the same concentration (32.5 μmol/l). In order to allow fast comparison of the individual spectra, all spectra were scaled in the way that the resonance of the standard had in all cases the same intensity. Although the PL concentrations are rather similar if the supernatant is considered, it is obvious that CHAPS band 1 did not contain significant amounts of lipids. It is also remarkable that only in the case of Triton X-100 phosphatidylinositol (PI) and a significant enrichment of SM and PE (cf. last column in Table 3) could be detected (particularly in the case of band 2) with reference to the concentrations of the individual PC species. Finally, the completely different amounts of the individual phospholipid species in the different fractions are remarkable.

**Figure 7:**
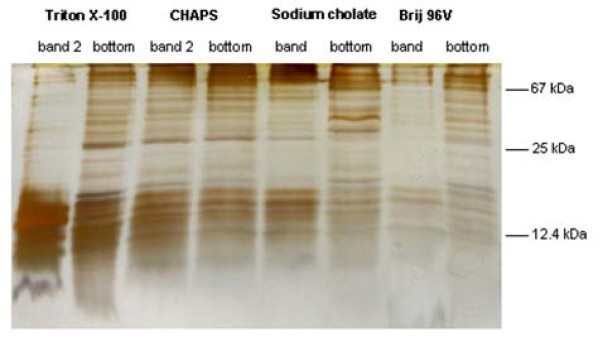
**PAGE of proteins (Silver staining) of the indicated band and bottom fractions from sucrose gradients after ultracentrifugation as described above**. The molecular wieghts of some selected proteins are indicated in the right part of the figure.

In addition to the lipids we also analysed the protein contents (cf. Figure 7) of the most prominent band fractions in comparison to the bottom fractions of the sucrose gradients. PAGE clearly indicated that after Triton X-100, sodium cholate and Brij 96V treatment only selected proteins are detectable in the individual band fractions. A massive accumulation of proteins with molecular weights smaller than 25 kDa could be observed in band 2 of the Triton X-100 sample. Although a more detailed analysis of the different protein bands was beyond the scope of this paper (but will be the topic of our future research), it is obvious that in addition to the lipid composition, the protein composition of the isolated DRMs is also strongly dependent on the applied detergent.

## Discussion

In the present paper we have characterized the disintegration of boar sperm cells by different detergents. The impacts of detergents on membrane lysis as well as lipid and protein compositions of solubilised and non-solubilised membrane fractions were investigated by different biochemical and biophysical methods. The detergents used here represent ionic and non-ionic compounds which are often used to study DRMs in biological and artificial membranes [6].

Although detergents may solubilise both, lipids and proteins from membranes, little attention was paid so far to cellular lipids from sperm and, thus, the focus of this paper was on the characterisation of lipids solubilised or non-solubilised by the different detergents. Because of the lipophilic properties of detergents, their potential interfering effects on lipid extraction and on the assays for lipid characterization had to be considered: Non-treated and detergent-treated whole sperm suspensions were extracted and analysed for their lipid contents. First, extraction efficiency and colorimetric lipid analyses were unaffected by the presence of detergents. Second, MALDI-TOF MS analyses could not be applied to organic sperm extracts after treatment with detergents because the lipid signals were strongly affected by the different detergents. This particularly holds for the ionic detergents such as sodium cholate or CHAPS and even applies to the organic extracts of DRM bands which were supposed to contain only marginal amounts of detergents. Third, in contrast to mass spectrometry, ^31^P NMR spectra were not affected by residual detergents. NMR analysis revealed no differences in the content and composition of lipids with and without detergent treatment. This is in accordance with the outcome of the colorimetric assays.

To elucidate the effect of detergents on sperm cell disintegration, light microscopy was performed so that membrane detachment - particularly from the acrosomal area of the sperm head - could be analysed. Notably, none of the detergents solubilised and detached membranes completely from the boar spermatozoa. Therefore, disintegrated or slacked membranes were mechanically removed. Subsequently, a centrifugation step separated cellular debris from supernatant containing released membranes of which the lipid and protein contents were determined. Without previous detergent treatment, the shearing forces alone did not cause a significant membrane loss from intact boar spermatozoa.

The detergents varied significantly in their impact on sperm cells with regard to disintegration, membrane detachment and extraction of lipids and proteins. In contrast to the other detergents, Pluronic F-127 which is a relatively mild detergent did not cause a detachment of membranes from the sperm head and did not remove significant amounts of lipid or protein. The determined protein and lipid concentrations were comparable to the control sperm sample without detergent. Pluronic F-127 consists of a very long sequence of bismethylene-oxy repeat units. Since this detergent is able to facilitate/mediate the entrance of non-permeable fluorescent dyes into spermatozoa or other cell types [37], we surmise that some molecules have few particular insertion sites into the membrane, but the remaining chains form a kind of a "protective shield" over the membrane. The permeabilisation of the bull sperm membrane is already achieved at lower concentrations of Pluronic F-127 (< 0.02%) [38]. Therefore it is an interesting outcome of this paper that the higher concentration of 2% did not boost the membrane disturbing effect.

Tween 20, a more lipophilic non-ionic detergent than Pluronic F-127, destabilised the sperm membranes. This destabilisation was enhanced with increasing temperature. Tween 20 consists of four long polyoxyether chains which probably serve as a lipid-like environment for proteins and which may explain its preferential use for protein extraction. Presumably due to its structure, insertion of Tween 20 into the sperm membranes forces the formation of droplets or micelles as observed by light and electron microscopy. The described membrane alterations affected both, head and tail membranes.

The most lipophilic non-ionic detergents Triton X-100 and Brij 96V show severe membrane disturbations and caused a significant loss of cholesterol, phospholipid and protein from the cells. The strongest detergent-induced release of cholesterol, phospholipid and protein was observed for the anionic sodium cholate and zwitterionic CHAPS. The acrosomal regions of the head and tail membranes were solubilised as confirmed by electron microscopy. Membrane disturbance was largely extended to the nuclear and mitochondrial membranes. Regarding these four detergents, the extent of membrane disintegration was lower in the postacrosomal area. Cholesterol and phospholipids were removed to about the same percentage from the cells; only after treatment with Triton X-100 a slightly smaller proportion of cellular cholesterol than phospholipid was found in the disintegrated fraction.

Only sodium cholate and Triton X-100 produced resolved and sharp bands on a sucrose gradient whereby only the band fractions of sodium cholate, CHAPS and Triton X-100 contained essential amounts of cholesterol, lipid and protein and were further analysed in more detail. Despite the rather low sensitivity of NMR spectroscopy, significant differences could be obtained upon the comparison of the band fractions: Only in the case of Triton X-100, an accumulation of phosphatidylinositol as well as sphingomyelin and - less pronounced - phosphatidylethanolamine could be monitored.

A high percentage of cholesterol compared to phospholipid was exclusively detectable in the band fractions of Triton X-100-treated sperm cells. Since it is known that cholesterol inhibits the solubilisation of phosphatidylcholine or phosphatidylethanolamine membranes by Triton X-100 [39] it can be assumed that Triton X-100 molecules were not inserted into existing cholesterol-rich membrane domains. Therefore, those "light" domains would be allowed to float within the gradient. Thus, only a small part of the cholesterol could be determined in the bottom fraction. Brij 96V which is structurally similar but more lipophilic than Triton X-100, solubilises sperm membranes in a different manner. However, only very little turbid detergent resistant material was collected from the top of the gradient (lowest density). The protein accumulation in this band was similar to the pattern found in band 2 after sperm treatment with Triton X-100.

Considering sodium cholate and CHAPS, the structural similarity to cholesterol implies their preferred insertion into membranes which could be modulated by ionic interactions. Membrane domains with inserted sodium cholate would also be expected to float since the molecular weight of the detergent is similar to cholesterol. This could explain why the bottom fraction of sodium cholate harboured only a small part of the underlayed cholesterol. In the case of CHAPS with higher molecular weight, one turbid band was visible just above the bottom fraction.

Summarising, the characteristic formation of turbid detergent-resistant band fractions with a relative accumulation of cholesterol, sphingomyelin, phosphatidylinositol and phosphatidylethanolamine was unique after treatment of boar sperm cells with Triton X-100. Moreover, band 2 after treatment of boar spermatozoa with Triton X-100 clearly showed the most pronounced selective accumulation of proteins, when compared with the bottom fraction and compared with the other detergents used. This may explain why this detergent was so far nearly exclusively used in the field of DRM research. Van Gestel [40] identified three differently glycosylated types of spermadhesin AQN-3 (< 20 kDa), superoxide dismutase and sp32 precursor as essential protein components of boar sperm DRMs after treatment with Triton X-100. We found proteins with a comparable molecular weight in DRMs solely after treatment with Triton X-100. Superoxide dismutase is involved in sperm protection against oxidative stress and sp32 is a proacrosin binding protein which is tyrosine phosphorylated during sperm capacitation [41]. AQN-3 belongs to seminal fluid proteins which cover spermatozoa and mediate the sperm interaction with the female genital tract and the oocyte (for review see [42]). The fact that monomeric or dimeric AQN-3 was retained on a phosphorylethanolamine affinity column [43] indicates a preferable association of this protein to phosphatidylethanolamine in DRMs. In accordance with that result we found an enrichment of phosphatidylethanolamine in Triton X-100 resistant fractions. Obviously, Triton X-100 has the most appropriate lipophilic properties allowing the study of important regulatory proteins in their functional lipid environment. Future analyses of those DRM-associated proteins in their lipid environment will contribute to the discussion about the natural existence of DRMs in dependence of cholesterol-related stabilisation and destabilisation of sperm membranes.

## Competing interests

The authors declare that they have no competing interests.

## Authors' contributions

UJ performed the most important cellular experiments and designed the study. UJ was supported by RS (MALDI-TOF MS of detergents), BB (protein analysis), GW (microscopy techniques) and BF (^31^P NMR analysis). KM performed the most important parts of data analysis. Finally, KM and JS wrote the manuscript and discussed the data.

## Supplementary Material

Additional file 1 **Table S1**. Survey of the individual detergents used in the present study, including their trade names, chemical structures, molecular masses and selected physico-chemical properties Click here for file
