# Supplementary material for: The solubilisation of boar sperm membranes by different detergents - a microscopic, MALDI-TOF MS, 31P NMR and PAGE study on membrane lysis, extraction efficiency, lipid and protein composition
Source: Lipids Health Dis. 2009 Nov 11;8:49. doi: 10.1186/1476-511X-8-49 (PMC2781011; doi:10.1186/1476-511X-8-49)
Supplement: Additional file 1 — Table S1. Survey of the individual detergents used in the present study, including their trade names, chemical structures, molecular masses and selected physico-chemical properties [file 1476-511X-8-49-S1.doc]

**Table S1:** Survey of the individual detergents used in the present study, including their trade names, chemical structures, molecular masses and selected physico-chemical properties

| **Detergent**  final concentration  [w/v] | **Charge** | **Critical Micellar Concentration in H_2_O [mM]** | **Hydrophilic Lipophilic Balance Number** | **(Mean) Molecular Mass [g · mol^‑1^]** | **Structure** |
| --- | --- | --- | --- | --- | --- |
| Pluronic F‑127  Sigma P2443 / 2% | nonionic | 4 to 11 | 18 bis 23 | 12600 | 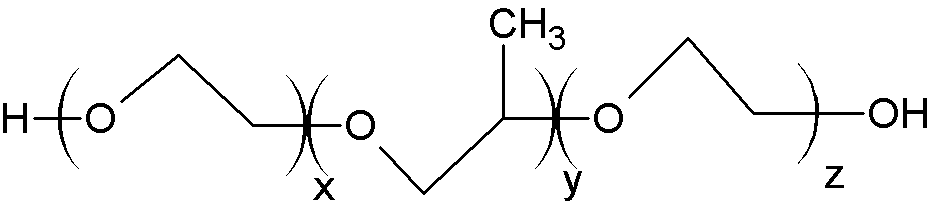 |
| Sodium cholate  Fluka 27029 / 1% | anionic | 9 to 15 | 18 | 431 | 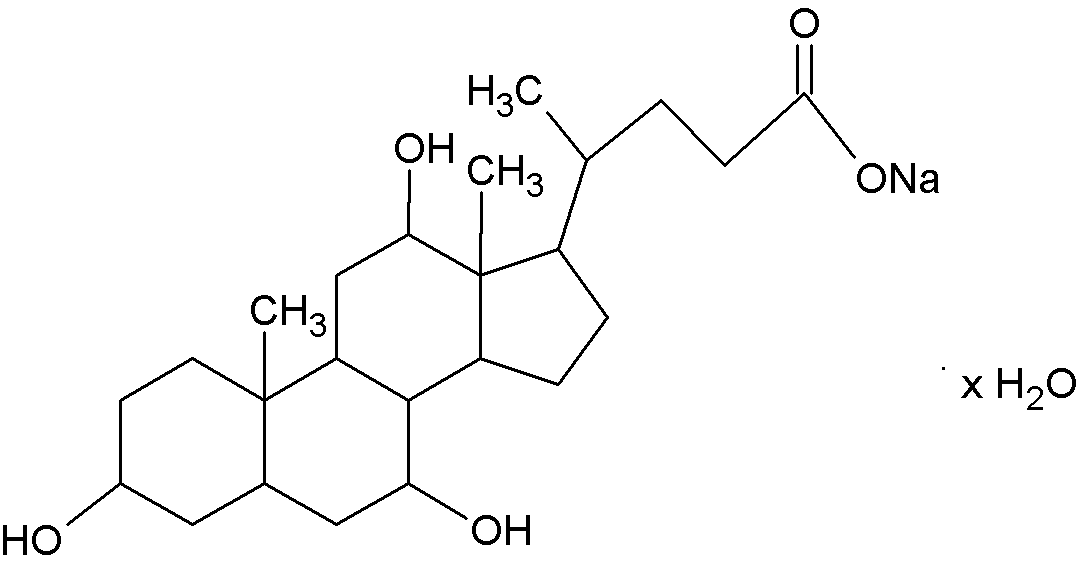 |
| CHAPS  Fluka 26680 / 4% | zwitter-ionic | 6 to 10 |  | 615 | 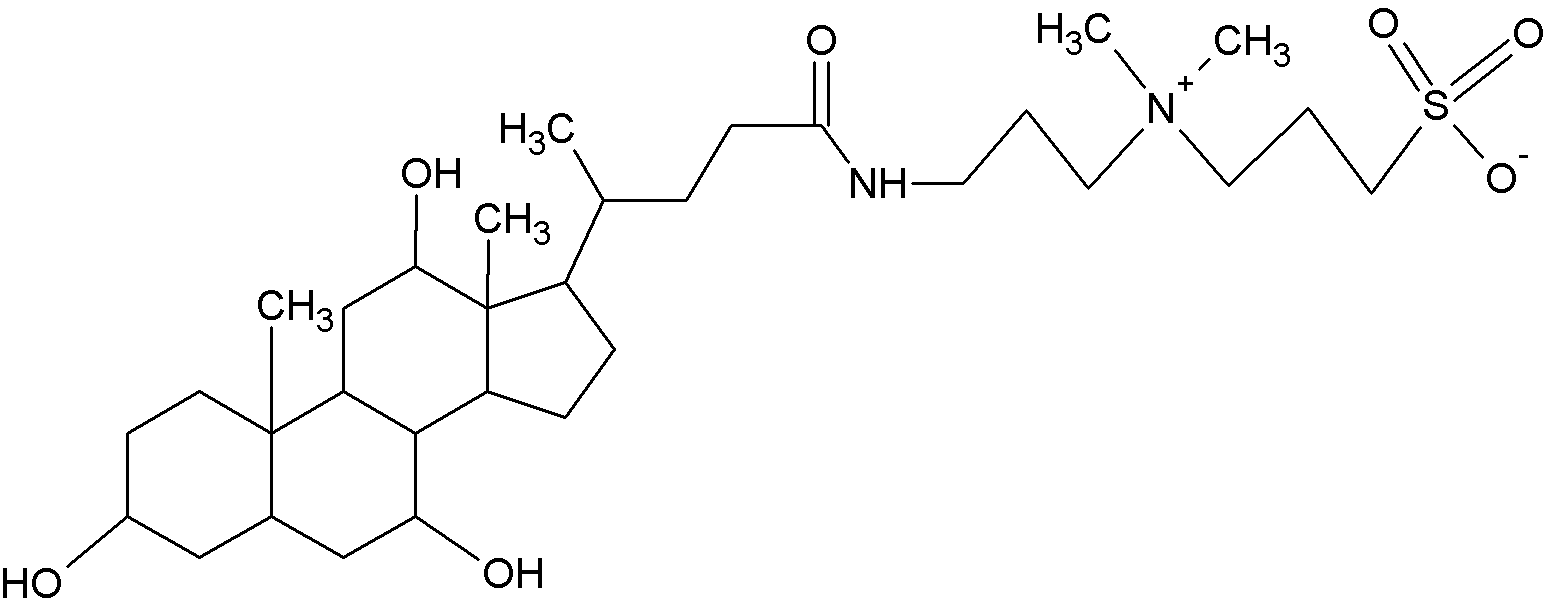 |
| Tween 20  Fluka 93773 / 1% | nonionic | 0,059 | 16,7 | 1228 | 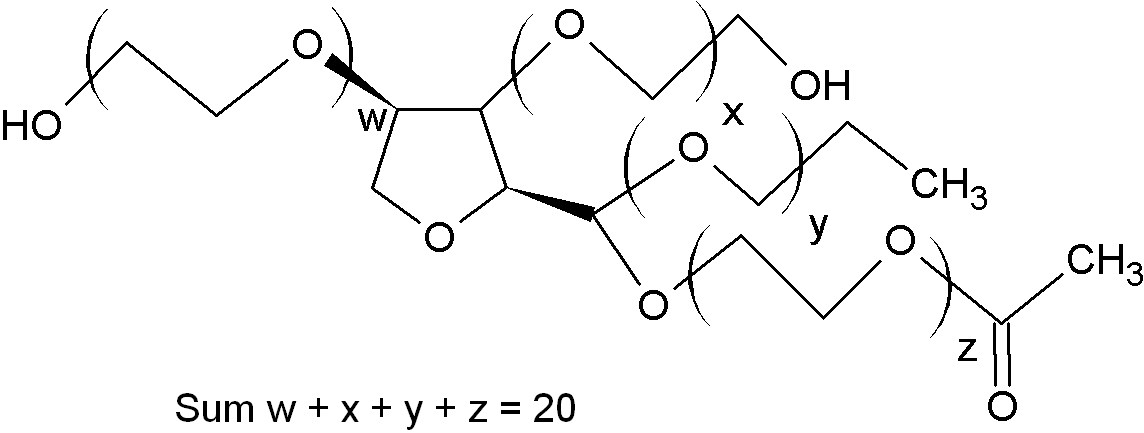 |
| Triton X‑100  Fluka 93426 / 1% | nonionic | 0,2 to 0,9 | 13,5 | 625 | 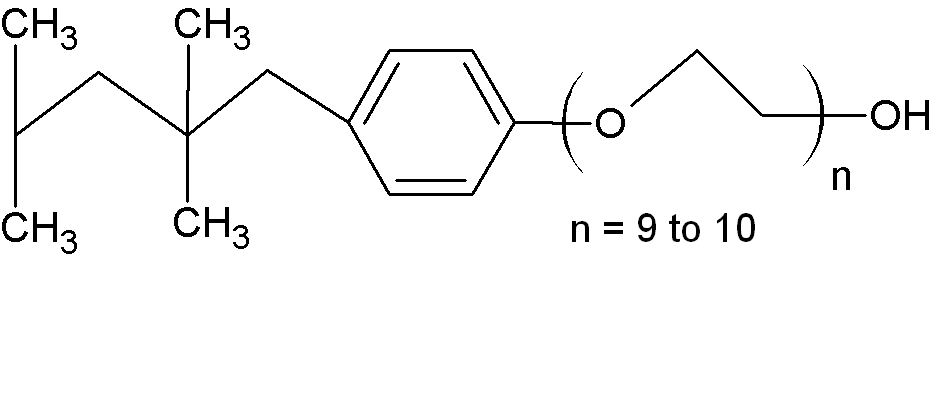 |
| Brij 96 V  Fluka 16011 / 1% | nonionic | 0,04 | 12,4 | 709 | 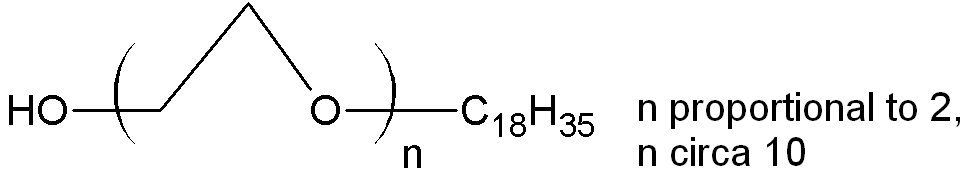 |

Please note that the formula and masses were calculated according to the data determined by positive ion MALDI-TOF mass spectrometry (see Results). Therefore, the provided data may be in slight contrast to the data provided by the suppliers of the individual detergents. Please also note that (in agreement with previous data (21)) several different species could be detected in the commercially available Tween 20 sample. However, only the most abundant species is shown.
